# One-Pot Conversion of Epoxidized Soybean Oil (ESO) into Soy-Based Polyurethanes by MoCl_2_O_2_ Catalysis

**DOI:** 10.3390/molecules22020333

**Published:** 2017-02-21

**Authors:** Vincenzo Pantone, Cosimo Annese, Caterina Fusco, Paola Fini, Angelo Nacci, Antonella Russo, Lucia D’Accolti

**Affiliations:** 1 Greenswitch s.r.l., 75013 Ferrandina (MT), Italy; enzopantone@hotmail.it (V.P.); antonella.russo@greenswitch.it (A.R.); 2 ICCOM-CNR, SS Bari, Via Orabona 4, 70126 Bari, Italy; annese@ba.iccom.cnr.it (C.A.); fusco@ba.iccom.cnr.it (C.F.); angelo.nacci@uniba.it (A.N.); 3 IPCF-CNR, SS Bari, via E. Orabona 4, 70125 Bari, Italy; p.fini@ba.ipcf.cnr.it; 4 Dipartimento di Chimica, Università di Bari “A. Moro”, Via Orabona 4, 70126 Bari, Italy

**Keywords:** catalysis, oxidation, bio-based polyurethane, one-pot synthesis

## Abstract

An innovative and eco-friendly one-pot synthesis of bio-based polyurethanes is proposed via the epoxy-ring opening of epoxidized soybean oil (ESO) with methanol, followed by the reaction of methoxy bio-polyols intermediates with 2,6-tolyl-diisocyanate (TDI). Both synthetic steps, methanolysis and polyurethane linkage formation, are promoted by a unique catalyst, molybdenum(VI) dichloride dioxide (MoCl_2_O_2_), which makes this procedure an efficient, cost-effective, and environmentally safer method amenable to industrial scale-up.

## 1. Introduction

In an era facing the depletion of fossil fuels and the increasing environmental concerns related to their burning, much of the current efforts have been directed to the challenging search for more sustainable sources that can ensure continuous manufacture of those commodities that have improved the quality of human life. As an example, recent years have witnessed a rapid increase in the production of bio-based plastics and polymers, entirely derived from renewable sources, such as starch, cellulose, sugars, etc. [1,2]. In this context, vegetable oils, such as ricinoleic, linseed, soybean oils, etc., have also been regarded as convenient source of renewable feedstocks to be employed in the development of bio-based polyurethanes (PU) [3,4,5,6,7,8,9].

Bio-based polyurethanes are available in a wide range of hardnesses, as well as in several formulations, to obtain different materials that allow energy savings, protect the environment, enhance safety, lead to innovative building systems, as well as several polyurethanes that have been studied for their preparation of composites [1,10,11,12,13].

**Scheme 1:**
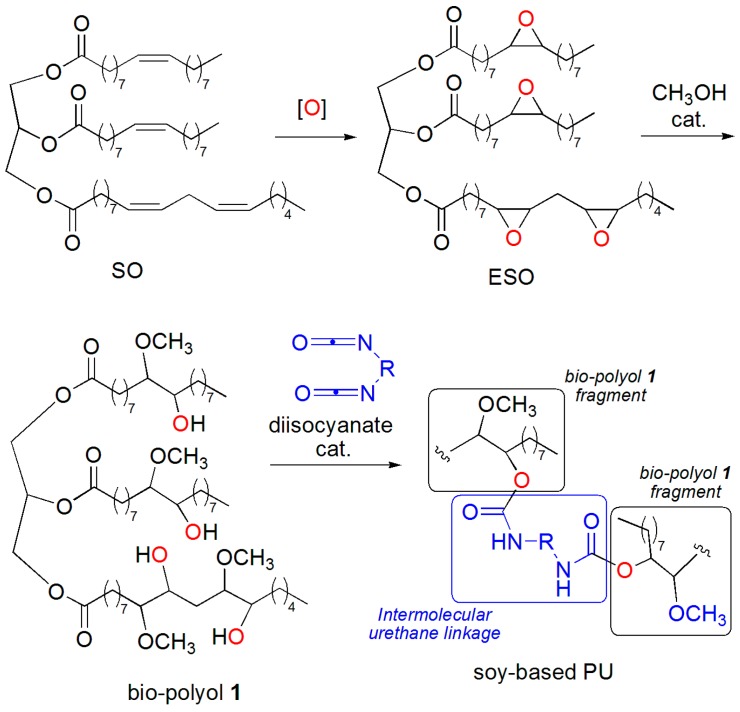
Representation of a typical chemical approach to the conversion of soybean oil (SO) into bio-based PU. For simplicity, only one of the possible regioisomers of soy-methanol polyol 1 is shown.

A practical approach to PU from vegetable oils involves epoxidation of the carbon-carbon double bonds of unsaturated fatty ester moieties and subsequent epoxide ring-opening reaction by nucleophilic reagents, providing the hydroxyl functionalities that form the urethane network upon reaction with isocyanates [3]. This is exemplified by the conversion of soybean oil (SO) into soy-based PU, through preliminary epoxidation, followed by methanolysis of the epoxidized oil (ESO), and reaction of the ensuing soy-methanol polyol 1 with suitable diisocyanates (Scheme 1).

In this context, special attention has been paid to oxidation methods employing organocatalysts [14,15] or based on mild and safe oxidants, such as H_2_O_2_ [16,17] organic (hydro)peroxides [18,19,20] and even O_2_ [21]. On the other hand, typical protocols prescribe the use of strong Brønsted acids (e.g., *p*-Toluene sulfonic acid SA, H_3_PO_4_, and HBF_4_) to catalyze the epoxide ring-opening reaction step, while hazardous Lewis acids (e.g., tin-, mercury-compounds) and/or toxic bases (e.g., tertiary amines) are usually employed in the formation of urethane linkages [22,23,24].

In our ongoing efforts aimed at searching new green catalytic methods [25,26,27,28] our interest in the area is driven by the search for an efficient, cost-effective, and environmentally safer conversion of epoxidized soybean oil (ESO) into bio-polyol 1 and then into soy-based PU (Scheme 1), with the ultimate goal of moving the whole process from laboratory bench to a pilot scale and then on to a manufacturing scale. Bio-polyol 1 shows interesting physico-chemical properties that makes it an excellent substitute for petrochemical polyols in the manufacture of some soy-based PUs.

In recent years, polyurethanes have been prepared by reacting a soybean oil-based polyol with different isocyanates to give foams, elastomers, coatings, and adhesives [1,8] in addition, the influence of a catalysts on gelling and blowing reactions was studied to compare the properties of polymers [22,23,24].

By taking advantage of the high versatility of dichlorodioxo-molybdenum(VI), MoCl_2_O_2_, as well as Lewis acid catalyst [29], this work describes our efforts in applying MoCl_2_O_2_ to the direct conversion of epoxidized soybean oil (ESO) into PU in a one-pot reaction that encompasses both the epoxide ring-opening stage and the subsequent urethane linkage formation step. In this way, the additional steps of acid catalyst neutralization and removal of the ensuing salts are completely bypassed. This, coupled with high efficiency and better environmental acceptance of MoCl_2_O_2_ over other metal-based catalysts, makes the whole process amenable to industrial scale-up.

## 2. Results and Discussion

Molybdenum is much less toxic than typical heavy metals. For this reason, molybdenum-based compounds have found several applications as clean alternatives to heavy metals in the petroleum and plastic industries. In particular, MoCl_2_O_2_, and its related complexes, serve well as Lewis acid catalysts for several organic transformations [29], ranging from oxidation to acylation, reduction, and carbamylation [30,31,32]

Reportedly, MoCl_2_O_2_ can efficiently catalyze the methanolysis of a range of terminal epoxides to β-methoxy alcohols, for which 5 mol % of catalyst generally suffices to drive reactions to completion within 1–5 h at 50 °C or, in some cases, even at room temperature [33].

**Scheme 2:**
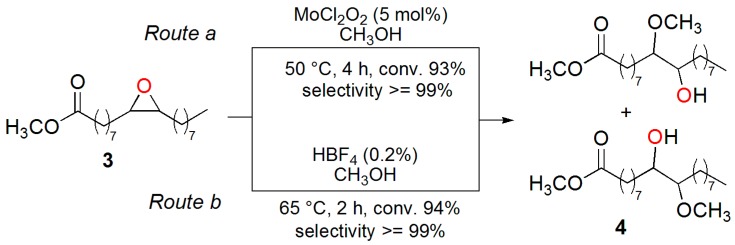
MoCl_2_O_2_-(route a) vs. HBF_4_-catalyzed (route b) methanolysis of methyl oleate epoxide (**3**) to β-methoxy alcohol **4**.

In order to test whether this protocol could be extended to the methanolysis of such internal epoxides as those of ESO (Scheme 1), we deemed it useful to first study the MoCl_2_O_2_-catalyzed epoxide-ring opening reaction of methyl oleate epoxide (**3**) with methanol under the reported conditions. To this purpose, methyl oleate epoxide (**3**) was prepared by reaction of commercial methyl oleate (**2**) with H_2_O_2_/formic acid, according to a widely-exploited industrial protocol [17]. Thus, upon treatment of epoxide **3** with 5 mol % MoCl_2_O_2_ in CH_3_OH at 50 °C (Scheme 2, route a), GC-MS analysis of the reaction mixture revealed that substrate conversion reached 93% after 4 h. Additionally, the MS and spectral data of the crude product displayed the characteristic features of methyl 10(9)-hydroxy-9(10)-methoxy-octadecanoate **4**, which was obtained with selectivity ≥99% as a roughly equimolar mixture of regioisomers. For unambiguous identification of β-methoxy alcohol **4**, a reference sample was made available upon subjecting methyl oleate epoxide (**3**) to conventional methanolysis by tetrafluoroboric acid (HBF_4_) catalysis, according to route b of Scheme 2 [34,35,36].

**Figure 1:**
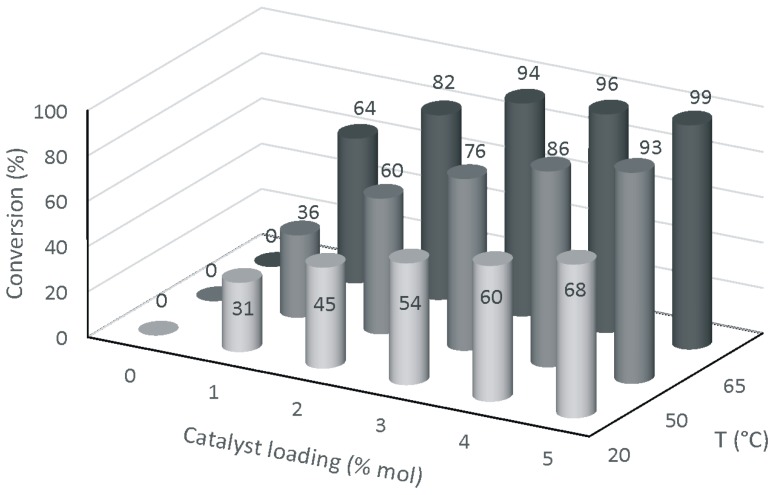
Diagram of the substrate conversion (%) vs. catalyst (MoO_2_Cl_2_) loading and temperature in the methanolysis of methyl oleate **3**.

Next, we turned to check whether the reaction conditions adopted were optimal for the case at hand. Thus, the reaction was studied under variable conditions of catalyst loading (1–5 mol %) and temperature (20–65 °C) at the fixed reaction time of 4 h. Results are collected in the diagram of Figure 1.

As shown in Figure 1, substrate conversion is directly correlated to catalyst loading and temperature. The GC-MS analysis of the reaction mixtures revealed that, in the range of temperatures and catalyst loading explored, product selectivity was retained, being always higher than 99%. Overall, these results point out that an increase in temperature can compensate for the decrease of catalyst loading, without significantly affecting product selectivity. This means that as long as the temperature is increased, the amount of catalyst can be reduced, which is often the most desirable condition for the development of economical processes. For instance, using 3 mol % of MoCl_2_O_2_ at 65 °C allows reaction to reach 94% conversion after 4 h (Figure 1), which is the same result as when the methanolysis is carried out under typical conditions (Scheme 2, route b).

**Figure 2:**
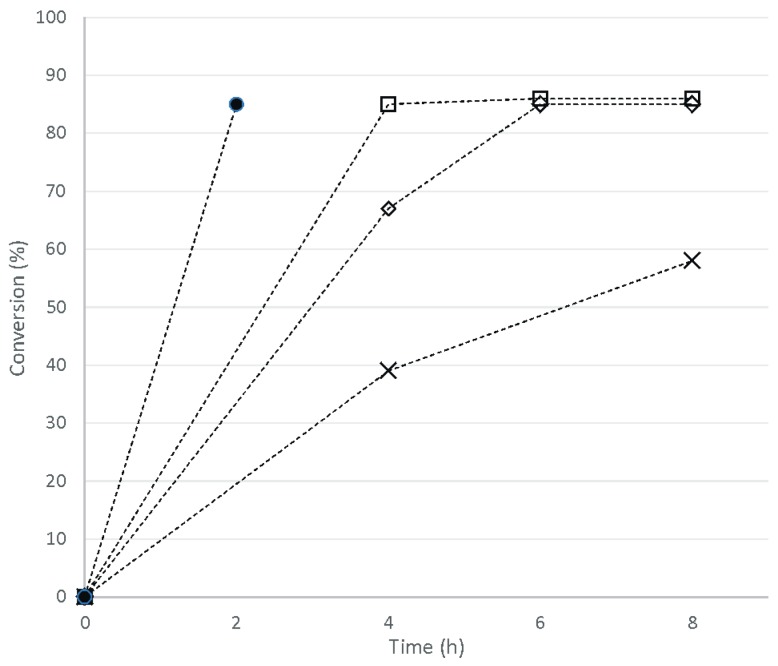
Conversion of substrate as function of time (h) and catalyst HBF_4_ (●) or MoCl_2_O_2_ (1 mol % (×), 2 mol % (◊), 3 mol % (□)) in the methanolysis of ESO.

**Figure 3:**
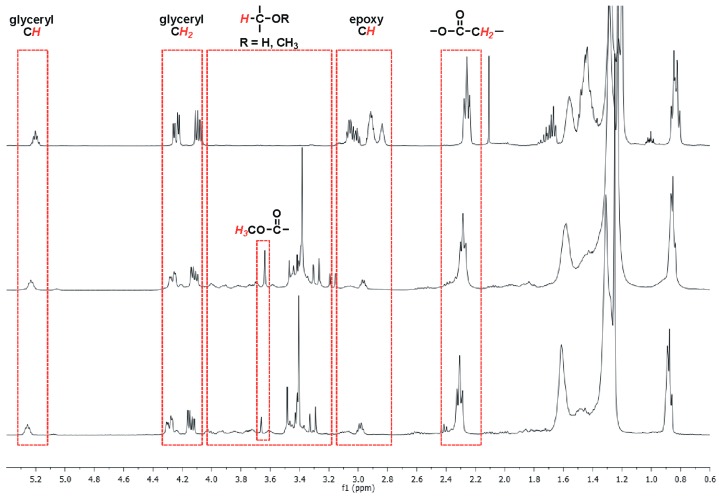
^1^H-NMR (CDCl_3_, 400 MHz) spectrum of (**a**) ESO; (**b**) bio-polyol **1** from HBF_4_-catalyzed methanolysis of ESO at 2 h, and (**c**) from MoCl_2_O_2_-catalyzed methanolysis of ESO at 2 h.

In light of these results, MoCl_2_O_2_ appears well-suited to be employed in the methanolysis of epoxidized fatty esters, as an alternative to HBF_4_. Therefore, we turned to explore its catalytic performance in the epoxide ring-opening reaction of ESO by methanol. Instructed by the new experimental conditions found for the methanolysis of methyl oleate epoxide (Figure 1), we performed all experiments at 65 °C, while catalyst loading was varied from 1 up to 3 mol %. ^1^H-NMR analysis of reaction mixtures was preferred over GC-MS techniques, allowing us to estimate substrate conversion and product identity, using the reference spectral data of bio-polyol **1** as obtained from typical HBF_4_-catalyzed methanolysis of ESO at 65 °C for 2 h [36,37]. Shown in Figure 2, methanolysis of ESO catalyzed by HBF_4_ attains a maximum of substrate conversion of 85% at 2 h, as estimated based on signal integration of residual epoxide protons (3.15–2.74 ppm) in the ^1^H-NMR spectrum of bio-polyol **1** (Figure 3b) in comparison with the corresponding signal integrations in the spectrum of ESO (Figure 3a). The singlet at 3.65 ppm in the spectrum of Figure 3b can be assigned to the resonance of methoxyl protons of fatty acid methyl esters (~7%), suggesting that transesterification is concomitant to the epoxide ring-opening reaction. Yet, it is seen that longer reaction times cause transesterification of triglycerides to take place at a rate of approximately 2% of fatty acid methyl esters formed per each additional hour of reaction.

Figure 2 shows that, in the MoCl_2_O_2_-catalyzed methanolysis of ESO at 65 °C, the maximum substrate conversion achievable is still as high as 85%–86%, regardless of catalyst loading and reaction time. However, the use of 1 mol % of MoCl_2_O_2_ requires as long as 24 h to allow conversion to reach 80%, while on doubling or tripling the catalyst concentration, 85% conversion can be smoothly reached within 4 or 6 h, respectively.

In all of the cases examined, reactions led to the selective conversion of ESO into bio-polyol **1**, as one can judge from the comparison of ^1^H-NMR spectral profiles of **1** obtained by HBF_4_ (Figure 3b) or MoCl_2_O_2_ (2 mol %, Figure 3c). In the ^1^H-NMR spectrum of Figure 3c, the signal at 3.65 ppm indicates the presence of minor amounts (~3%) of methyl ester side products. Unlike the case with HBF_4_ catalyst, their amount was found to not increase appreciably for prolonged reaction times, consistent with the weak propensity of MoCl_2_O_2_ to catalyze transesterification reactions [31,38].

Other hints of the conversion of ESO into bio-polyol **1** in the reaction with CH_3_OH/MoCl_2_O_2_ come from the measurement of physicochemical properties, such as density, viscosity, and the average number of hydroxyl and epoxy functionalities (Table 1).

Data reported in Table 1 indicate that it is possible to obtain the bio-polyol **1** with 3.45 hydroxyl groups per molecule, instead of 4, as would expect based on the average number of epoxy groups. This discrepancy has already been explained as result of the presence of ethereal bonds due to concurrent intramolecular epoxide ring-opening (cross-linking process).

**Scheme 3:**
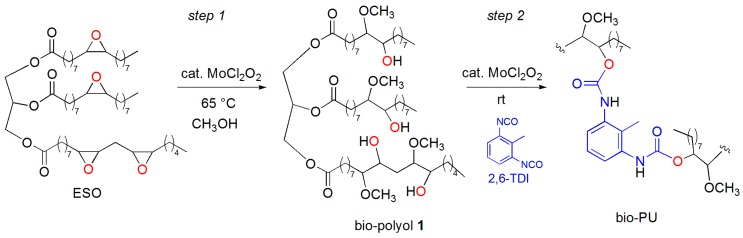
One-Pot preparation of soy-based bio-PU from ESO by means of MoCl_2_O_2_ catalysis.

Next, we turned our attention to the “step 2” of the one-pot MoCl_2_O_2_-catalyzed soy-based polyurethane production, i.e., the reaction of bio-polyol **1** with toluene 2,6-diisocyanate (2,6-TDI) (Scheme 3).

Brückner and coworkers have recently studied catalytic activity of MoCl_2_O_2_ and related complexes in the formation of mono-, di-, tri-, and tetracarbamates from a variety of alcohols and isocyanates [30]. They found that these reactions proceed to completion at room temperature in few minutes using as little as 0.1–1 mol % of the catalyst in chlorinated solvents.

Polyurethanes were prepared according to a reported procedure in the experimental section, where each polyol obtained by methanolysis reaction with 1–5 mol % of catalyst on respect to epoxide, after evaporation in vacuo of methanol, was mixed in a plastic cup with 2,6-TDI for 30 s using a high-speed mixer. The gel times at room temperature for each compound were 4–12 min.

Polyurethanes obtained from the polyols were quite similar, which was reflected in their physical and mechanical properties.

**Figure 4:**
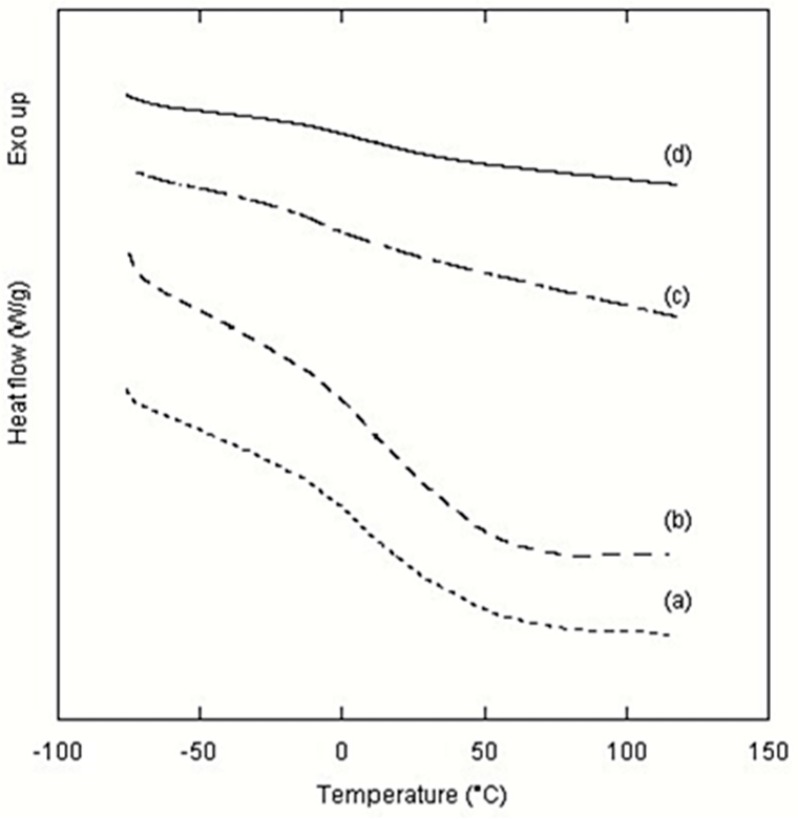
DSC curves for bio-PU made with bio-polyol **1** and (**a**) 1 mol %; (**b**) 2 mol %; (**c**) 3 mol % and (**d**) 5 mol % catalysts MoCl_2_O_2_.

The thermal properties of polyurethane samples, obtained using different amounts of catalyst, were analyzed by DSC seven days after their synthesis (Figure 4). No thermal evidence of melting, crystallization or decomposition were detected in the studied range of temperature from −80 °C to 120 °C. It turned out that all samples were in an amorphous state and thermally stable.

Thermal events attributable to glass transitions were observed only for samples prepared at lower catalyst quantity, 1 mol % and 2 mol %, with midpoints in the range between 9.4 and 10.5 °C (Figure 4a,b). These glass transition temperatures (*T*g) indicate that polyurethane samples prepared using the one-pot synthesis herein reported with 1 mol % and 2 mol % of catalyst are rubbery at room temperature.

As a control experiment, we prepared the same bio-PU using the literature procedure [36] based on the thermal catalysis. In this case, heating of the mixture for 24 h at 110 °C was necessary to complete the polymerization reaction, and a rubbery polymer with a *T*g of 20 °C was obtained.

Another piece of evidence supporting the suitability of the proposed method was obtained by polymerizing bio-polyol **1**, achieved with the HBF_4_, in the presence of fresh 2 mol % of MoCl_2_O_2_. The PU thus obtained displayed a *T*g value very close to that of the polymer achieved using the one-pot method.

**Figure 5:**
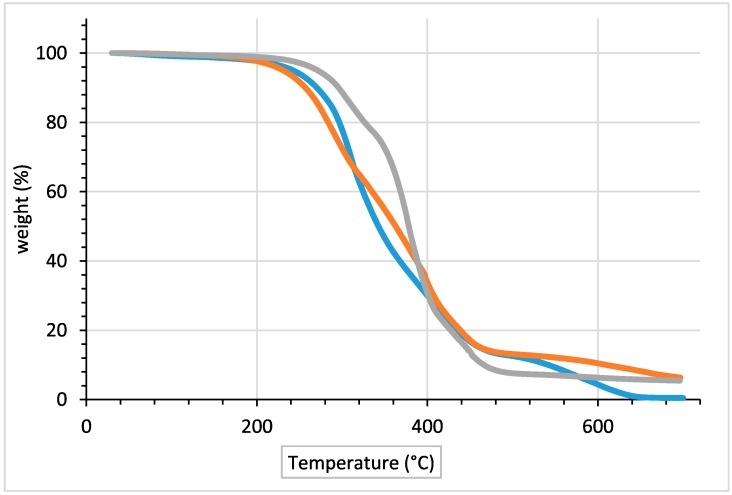
TGA curves for bio-PU made with bio-polyol **1** and 1 mol % (blue color), 2 mol % (red color) MoO_2_Cl_2_ catalysts and literature procedure (grey color) [36].

TGA curves of polyurethane samples obtained using 1 mol % and 2 mol % of Mo catalyst and literature procedure, analyzed by the first derivative (DTG), showed a multistep decomposition pattern in agreements with literature (Figure 5) [40,41,42,43]. At temperature lower than 200 °C the weight loss was due to small molecule products (not reacted isocyanates, water, ethers, etc.).

The urethane bond groups break up into isocyanates and polyols from 200 °C. At the same time, the polyols segments decompose to same kinds of aliphatic alcohols and the products become more complex as the evolved products interact with each other. At temperature above 350 °C, primary and secondary amines, vinyl ethers, CO_2_, HCN, and nitriles are the decomposition products [42,43].

The values of degradation temperatures for 5% weight loss (T_d5%_), for 70% weight loss (T_d70%_), for 90% weight loss (T_d90%_), the maximum degradation temperature (Tmax) and the char yield at 700 °C are shown in Table 2.

These results indicate that the polyurethane sample prepared using the literature procedure is initially the most stable. In fact, the 5% degradation weight loss is obtained at 272.1 °C, thirty and forty degrees above the weight loss temperatures observed for Bio-PU samples deriving from 1 mol % and 2 mol % catalyst. Moreover, temperatures at which the degradation process shows the maximum rate follow the same order: 291.7 °C for 2 mol % Bio-PU sample, 312.4 °C for 1 mol % Bio-PU sample and 378.3 °C for literature samples.

At about the 70% weight loss the situation changes. The literature polyurethane sample becomes less stable than polyurethane samples prepared using the one-pot synthesis herein reported. In particular, the Bio-PU with 2 mol % MoCl_2_O_2_ catalyst has the highest temperature at which the 90% degradations weight loss is obtained. However, the shapes of the weight loss curves of all polyurethanes are almost identical, and overall differences in thermal stability appear to be small [44,45].

## 3. Experimental Section

### 3.1. Material and Methods

NMR spectra were recorded on a Varian Inova 400 MHz (Agilent company, Santa Clara, CA, USA) or on an Agilent Technologies 500 MHz spectrometer (Agilent company); the ^1^H-NMR spectra (400 MHz) were referenced to residual isotopic impurity of CDCl_3_ (7.26 ppm). GC/MS experiments were run on a Shimadzu GLC 17-A instrument connected with a Shimadzu ITALIA GLC/MS QP5050A selective mass detector (Shimadzu, Milan, Italy) using a SLB-5MS column (30 m × 0.25 mm id, film thickness 0.25 μm). Mass spectra were performed in EI mode (70 eV). FTIR spectra (Perkin Elmer, Milan, Italy) are relative to KBr pellets or films (deposited on KBr plates).

Acetone and other common solvents were purified by standard methods. Commercial starting materials were purchased in highest purity available from scientific chemicals suppliers (Aldrich, Milan, Italy) and were used without purification. Soybean oil was provided by Valsoia (iodine number of 130 g I_2_ /100 g).

A Viscometer Cannon-Fenske reverse flows YNT instrument (Koehlerinstrument, NY, USA) was used to determine the viscosity using the standard method ASTM D445 (Standard Test Methods for Kinematic Viscosity of Transparent and Opaque Liquids). The determination of density was obtained with the pycnometer method EN ISO 1675.

Iodine number value, oxirane number (ON) and hydroxyl number of pre-polymers were determined by EN ISO 661, ASTM D 1652, and ASTM D4274 methods, respectively [36,37].

### 3.2. Synthesis of the Epoxide of Methyl Oleate *(**3**)*

The epoxide was prepared using H_2_O_2_/HCOOH and phosphoric acid as catalyst. To a stirred solution of methyl oleate (**2**) (34.6 g) at 60 °C, and 75% aqueous phosphoric acid (1.14 g) was added 50% hydrogen peroxide, H_2_O_2_ (18.11 g), in mixture with 85% formic acid (2.53 g) at regular intervals over two h. Then, the solution was heated at 75 °C and left to react for two h. Next, the temperature was lowered to 64 °C and 1.85 g of aqueous NaOH (24.9%) was added dropwise during 1 h, and the temperature slowly raised to 85 °C. Then, oxalic acid (0.12 g) was added and the mixture was left to react for 30 min. Next, reaction mixture was transferred into a separating funnel, and layered for 1 h. Finally, the aqueous phase was removed and the epoxide **3** was dried in vacuo at 98 °C for 1 h. Chemical properties of **3** were reported: number of oxirane oxygen: 5.3 g O_2_/100 g; number of epoxy groups: 1 per molecule; and amount of iodine: 1.1 g I_2_/100 g. The spectral data of epoxide **3** was in agreement with the literature [34,35].

### 3.3. Synthesis of 10(9)-Hydroxy-9(10)-methoxy-octadecanoate *(**4**)*

Starting from the corresponding epoxides, methanolysis reaction was performed using HBF_4_ as the catalyst, following a procedure from the literature [36,37]. The molar ratio of epoxy groups to OH was 1:11. The catalyst loading was 0.2% with respect to the total weight of the reaction mixture. Chemical properties of methyl 10(9)-hydroxy-9(10)-methoxy-octadecanoate (**4**) were reported: the number of epoxy groups: 0.03 per molecule; the amount of –OH: 161 mg KOH/g; and fn (hydroxyl functionality/molecule): 1. The spectral data of **4** were in agreement with the literature [34,35,37].

### 3.4. Synthesis of ESO

To a stirred solution of soybean oil (110.31 g) and 75% aqueous phosphoric acid (1.21 g) heated at 63 °C, a mixture of 50% hydrogen peroxide H_2_O_2_ (71.31 g) and 85% formic acid (9.89 g) was added at regular intervals of 4 h. Then, the solution was warmed at 75 °C for 2 h and, subsequently, an aqueous solution of NaOH at 25% was added dropwise for 1 h while the temperature was raised slowly up to 85 °C. Then, oxalic acid (0.29 g) was added and the mixture was left to react for 30 min. Then, the mixture was transferred into a separating funnel, and layered for 2 h. Finally, the aqueous phase was removed and the ESO was dried in vacuo at 98 °C for 1 h. The spectral data of ESO were in agreement with the literature [36] and with an authentic sample provided by Greenswitch Industry (Greenswicht, Ferrandina (MT), Italy).

Chemical properties of ESO were reported: amount of oxirane oxygen: 6.3 g O_2_/100 g; number of epoxy groups: 4 per molecule; amount of iodine: 1.5 g I_2_/100 g; and the number of double bonds/molecule: 0.05.

### 3.5. Methanolysis of ESO Using HBF_4_

Methanol 151.10 g (4.72 mol) and 1.11 g of 48% aqueous solution of HBF_4_ (0.006 mol) were added to a 500-mL, three-necked flask equipped with a refluxing column, a mechanical stirrer, and a thermometer [14]. To the refluxing mixture, heated with a water bath, 97.80 g of ESO (oxirane oxygen 6.3 g O_2_/100 g) were added and left to react for 2 h. After cooling to room temperature, aqueous ammonia (30%) was added to neutralize the catalyst and avoid hydroxylation. The mixture was washed with water and then purified on a rotary evaporator under a low vacuum at 98 °C for 1.5 h [14], yielding 102 g of bio-polyol **1**. Finally, the E-factor was determined using the Sheldon rules. [46,47].

#### Estimation of the E-factor:

Mass of reactants: 48% aqueous HBF_4_: 0.5328 g (solvent (water) has been excluded from this calculation); 30% aqueous NH_3_: 0.225 g (solvent (water) has been excluded from this calculation); CH_3_OH: 151.1 g; ESO: 97.8 g; total amount of reactants: 0.5328 g + 0.225 g + 151.1 g + 97.8 g = 249.6578 g.

Mass of product: bio-polyol **1**: 102 g; total amount of final products: 102 g.

Amount of waste: (249.6578 − 102): 147.6578 g.

E-Factor = Amount of waste/Amount of product = 147.6578/102 = 1.45.

### 3.6. Synthesis of Methyl 10(9)-Hydroxy-9(10)-methoxy-octadecanoate *(**4**)* Using MoCl_2_O_2_

The following procedure was representative for methanolysis of **3**. To a stirred solution of methyl oleate epoxide (**3**) (1.0 g, oxirane oxygen value with 5.3 g O_2_/100 g) in methanol (1.2 g, 37.5 mmol) heated at 65 °C, 32 mg of MoCl_2_O_2_ (5 mol % respect to epoxide) were added in one portion. The reaction progress was monitored by GC/MS. After 4 h (conv. 98%), the solvent was removed in vacuo at 98 °C for 1.5 h, thus obtaining methyl 10(9)-hydroxy-9(10)-methoxy-octadecanoate (**4**) [34,35] as a colorless oil (number of oxirane oxygen: 0.04 g O_2_/100 g; number of epoxy groups: 0.02 per molecule; amount of –OH: 164 mg KOH/g; fn (hydroxyl functionality/molecule): 1).

### 3.7. Methanolysis of ESO Using MoCl_2_O_2_

To a stirred solution of ESO (1.0 g, oxirane oxygen value 6.3 g O_2_/100 g) in methanol (1.4 g, 40 mmol) heated at 50 °C, 8.48–42.3 mg of MoCl_2_O_2_ (1–5 mol % with respect to epoxide) were added in one portion. The reaction progress was monitored by ^1^H-NMR. After 4 h (conv. 98%), the solvent was removed in vacuo at 98 °C for 1.5 h, thus obtaining bio-polyol **1** (1.11 g); amount of OH: 191 mg KOH/g; fn (hydroxyl functionality/molecule): 3.45; number of epoxy groups: 0.03 per molecule; oxirane oxygen value: 0.05; density at 25 °C: 1.001761 (kg/dm^3^); kinematic viscosity at 25 °C: 4543 (cSt); dynamic viscosity at 25 °C: 4551 (cP) [12]. The same reaction was repeated with 100 g of ESO, yielding 110 g of bio-polyol **1**, and 84 g of methanol was recovered using the column with structured packing MellapakPlus™. The E-factor was determined using the Sheldon rules. [46,47].

#### Estimation of the E-Factor:

Mass of reactants: MoCl_2_O_2_: 1.7 g (2 mol %); CH_3_OH: 140 g; ESO: 100 g; total amount of reactants: 1.7g + 140 g + 100 g = 241.7 g.

Mass of products: bio-polyol **1**: 110 g; MoCl_2_O_2_: 1.70 g; CH_3_OH: 84 g; total amount of final product: 110 g + 1.70 g + 84 g = 195.7 g.

Amount of waste: (241.7 − 195.7) = 46 g.

E-Factor = amount of waste/amount of product = 46/195.7 = 0.24.

### 3.8. One-Pot Synthesis of Bio-Based PU

The consecutive steps of methanolysis of ESO and the reaction of the polyolic products with 2,6-TDI were performed in a one-pot manner as follows: (a) methanolysis of ESO according to the previous procedure using the desired MoCl_2_O_2_ catalyst concentration (1–5 mol %); (b) reaction of the as prepared bio-polyol **1** (containing the catalyst) by mixing with 2,6-TDI at room temperature in a -NCO/-OH molar ratio of 1.1: 1. Reaction mixture was left to react at room temperature, without stirring, for 24 h, yielding the Bio-PU, which was characterized by DSC (Q200 TA Instruments, Perkin Elmer, Milan, Italy) and TGA (Pyris 1 Perkin Elmer, Milan, Italy) analysis under N_2_ atmosphere with heating rate of 20 °C/min (*T*g 10–12 °C are better evident in the second heating scan). The thermal analysis of all samples were performed 7 days after their synthesis in order to have consistent results.

### 3.9. Synthesis Bio-Based PU without Catalyst

Bio-PU was prepared using bio-polyol **1** and 2,6-TDI in a NCO/OH molar ratio of 1.1:1. The polyol and the isocyanate components were stirred for 2 min, then the mixture was poured into a mold and the unit was left in vacuo to evacuate bubbles (5 min at 60 °C). The sample was put into an oven for 24 h at 110 °C to complete the reaction. The sample was then cooled to room temperature and remolded. The *T*g after seven days was found to be equal to 20 °C, unlike what is reported in literature (ca. 50 °C) [37].

## 4. Conclusions

In conclusion, an atom-economical, cascade, one-pot, and two-step method for polyurethane synthesis has been developed using the inexpensive and environmentally friendly catalyst MoCl_2_O_2_, [10], as also confirmed by the safety data given in databases of ECHA (European CHemistry Agency) [48]. Physicochemical properties of PURs obtained with this method are quite similar to those of materials prepared by procedures in the literature.

Other important advantages of the present method are summarized as follows:

- The synthesis of PU is carried out in a short time and at room temperature, a great advantage when compared with reactions carried out without catalysts;
- The efficiency and selectivity of the methanolysis step provides a pure polyol intermediate, which could be used directly in subsequent reactions, avoiding the costly and time-consuming purification procedures that are necessary when using the classical method based on HBF_4_; and
- Methanol can be easily recovered and reused, thus increasing the atom economy of the process. The E-factor for these reactions is completely different [46,47] indeed, the MoCl_2_O_2_ allows the recovery of methanol (purity 99.9%), which can be recycled (see experimental section for each procedure).

**Figure 6:**
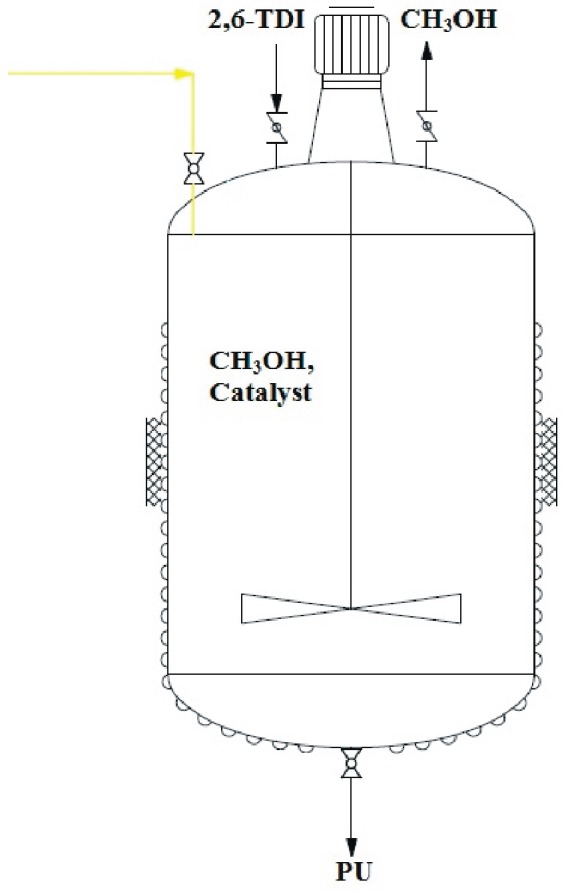
Pilot apparatus [49].

The final goal of this work is the construction of a pilot apparatus located at Greenswitch S.r.l. Company in Ferrandina (ITALY), of which only a part is shown in Figure 6 [49].

## Figures and Tables

**Table 1:** Physical and chemical characteristics of bio-polyol **1**.

#	Density (Kg/dm^3^)	Viscosity (cP)	n. OH ^a^ (mg of KOH/g)	n. OH/Molecule	n. Epoxy/Molecule	E-Factor ^b^
**1** from HBF_4_	1.001761	4551	191	3.45	----	1.45
**1** from MoCl_2_O_2_	1.003678	4567	188	3.40	----	0.24

^a^ mg of KOH required to neutralize 1 g of polyol treated with excess acetic anhydride, according to ASTM, D4274. For additional info, please refer [39] ^b^ See experimental section for calculation.

**Table 2:** The thermal properties of the polyurethane samples.

Material	T_d5%_ (°C)	T_max_ (°C)	T_d70%_ (°C)	T_d90%_ (°C)	M_residue_ (%)
Literature procedure	272.1	378.3	401.02	465.98	5.4
BIO PUR with 1 mol % MoCl_2_O_2_ catalyst	242.0	312.4	401.02	540.0	0.5
BIO PUR with 2 mol % MoCl_2_O_2_ catalyst	231.5	291.7	408.0	609.0	6.3
